# Preoperative ultrasound risk stratification of submandibular gland lesions: a retrospective cohort study

**DOI:** 10.3389/fsurg.2026.1869074

**Published:** 2026-08-12

**Authors:** Andreas Spörlein, Anna Michelle Flegler, Valentin Burkhardt, Tobias Schulz, Stefan Richtsfeld, Christoph Becker, Naglaa Mansour, Kathrin Gerstacker, Andreas Knopf

**Affiliations:** Department of Otorhinolaryngology – Head and Neck Surgery, Medical Center – University of Freiburg, Freiburg, Germany

**Keywords:** malignancy prediction, risk stratification, salivary gland tumors, submandibular gland, taller-than-wide, ultrasound

## Abstract

**Background:**

Submandibular gland lesions are rare and diagnostically challenging because benign neoplasms, inflammatory mass lesions, carcinomas, and lymphomas may present with overlapping sonographic features. While ultrasound is central to the preoperative work-up, most malignancy prediction models were developed in mixed major salivary gland cohorts dominated by parotid tumors. Their performance in the submandibular gland remains unclear.

**Methods:**

We conducted a retrospective cohort study of consecutive patients who underwent submandibulectomy for an intraglandular lesion at a tertiary referral center between 2019 and 2025. All patients had standardized preoperative ultrasound and histopathologic confirmation. Ultrasound images were reviewed while blinded to clinical and histopathological outcomes. Clinical variables, conventional sonographic features, the taller-than-wide (TTW) ratio, the score proposed by Lo et al., and a TTW-modified version of this score were evaluated for their ability to discriminate benign from malignant lesions.

**Results:**

A total of 120 patients were included (mean age 58.4 ± 17.4 years; 75% male). Final histology showed 96 benign lesions (80.0%), 13 carcinomas (10.8%), and 11 lymphomas (9.2%). Among clinical features, only nerve palsy was associated with malignancy (4.2% vs. 0%, *p* = 0.04). On ultrasound, malignant lesions more frequently showed irregular margins (37.5% vs. 17.7%, *p* = 0.04) and suspicious cervical lymph nodes (20.8% vs. 1.0%, *p* = 0.001). Other ultrasound features were not significantly different between benign and malignant lesions. The TTW ratio differed in exploratory subgroup analysis across benign tumors, carcinomas, and lymphomas (*p* = 0.02), but not in the overall benign-versus-malignant comparison. The Lo et al. score showed poor discrimination (AUC 0.62, 95%-CI 0.48–0.76), and the TTW-modified score performed similarly (AUC 0.61, 95%-CI 0.47–0.74).

**Conclusion:**

Preoperative ultrasound is valuable in the diagnostic assessment of submandibular gland lesions, particularly for identifying irregular margins and suspicious cervical lymph nodes, but morphology alone has limited ability to distinguish benign from malignant disease. Parotid-derived ultrasound risk scores, including TTW-based modification, showed poor transferability to the submandibular gland.

## 1 Introduction

Tumors of the submandibular gland are rare, accounting for approximately 5%–15% of salivary gland neoplasms and less than 2% of head and neck tumors (1–3). Compared with the parotid gland, submandibular gland tumors show a higher relative risk of malignancy, with many institutional series reporting that roughly one-third to one-half of submandibular gland neoplasms are malignant (2–5). This combination of rarity, histologic heterogeneity, and comparatively higher malignant potential makes accurate preoperative risk estimation particularly consequential for selecting biopsy technique, counseling patients, and planning surgery (2, 4).

The differential diagnosis of a submandibular triangle mass extends beyond primary submandibular gland tumors and includes inflammatory/obstructive salivary disease, benign neoplasms, primary carcinomas, metastatic disease, and lymphoproliferative disorders within or outside of the submandibular gland (6–8). Clinically, patients often present with a painless, slowly enlarging mass, while pain, rapid growth, or cranial nerve symptoms may raise suspicion of malignancy, yet these clinical cues are neither sufficiently sensitive nor specific to obviate imaging and tissue diagnosis (4, 9, 10).

Management paradigms depend fundamentally on whether the lesion is benign, malignant, or lymphoproliferative (7, 11, 12). For benign submandibular gland lesions, gland excision has historically been standard, while some have proposed gland-preserving approaches (13, 14). For malignant submandibular gland tumors, international guidelines emphasize surgery as the primary modality and recommend adjuvant radiotherapy for selected high-risk situations, while recognizing that evidence is often derived from mixed major salivary gland cohorts (15–17). Lymphoproliferative diseases of the submandibular gland, most commonly lymphomas (especially MALT lymphoma, diffuse large B-cell lymphoma, and follicular lymphoma), are typically treated with systemic therapy tailored to the specific lymphoma subtype rather than primary surgical excision (18).

High-resolution ultrasound (US) is central for preoperative risk stratification because the submandibular gland is superficially located, enabling detailed real-time assessment of gland parenchyma and adjacent cervical lymph nodes (19–21). Ultrasound can characterize lesion morphology (shape, margins, echotexture, calcifications) and vascular patterns using color Doppler (19–21). In addition, US enables fine-needle aspiration (FNA) sampling, which typically shows high specificity but moderate sensitivity for malignancy in the submandibular gland (16, 22–24), and core needle biopsy, which has higher sensitivity, but is more invasive (25). Frozen-section pathology can also be used effectively and helps guide the extent of surgery intraoperatively (26, 27).

Most of the evidence regarding submandibular gland ultrasound is based on general salivary gland cohorts with most tumors located in the parotid gland (19, 20), while studies specifically addressing the submandibular gland are scarce (7). This also affects ultrasound-based malignancy prediction, for example the model proposed by Lo et al. (28). Their multivariable model incorporates five readily assessed sonographic parameters—boundary, regional lymphadenopathy, shape, posterior acoustic enhancement, and calcification—and demonstrated high discrimination and specificity at the proposed cutoff in internal cross-validation, but has only been validated in a mixed cohort with 21.6% of tumors originating in the submandibular gland (28).

“Taller-than-wide” (TTW) orientation is a malignancy-associated feature that has been validated in thyroid and breast imaging and has recently been translated to parotid gland tumors (29). A TTW configuration occurred more frequently in malignant than in benign tumors, and a visually apparent TTW shape was highly specific for malignancy, albeit with limited sensitivity in a recent parotid cohort (29). Whether TTW provides incremental diagnostic value in submandibular gland tumors—where tissue planes, tumor biology, and the competing diagnosis of lymph node pathology differ—remains unknown.

Against this background, the present study aimed to evaluate the value of preoperative ultrasound for risk stratification of submandibular gland lesions in a surgically treated cohort with histopathologic confirmation. Specifically, we investigated whether ultrasound can reliably distinguish benign from malignant submandibular gland lesions, how established ultrasound-based risk stratification systems, including the model proposed by Lo et al., perform when applied specifically to this gland, and whether the taller-than-wide criterion contributes diagnostically in the submandibular setting. In contrast to previous studies focusing *post hoc* on resected submandibular tumors, our study applies a lesion-centered approach that also includes inflammatory disease presenting as demarcated lesions and therefore better reflects the clinical reality of preoperative ultrasound assessment.

## 2 Materials and methods

**Figure 1:**
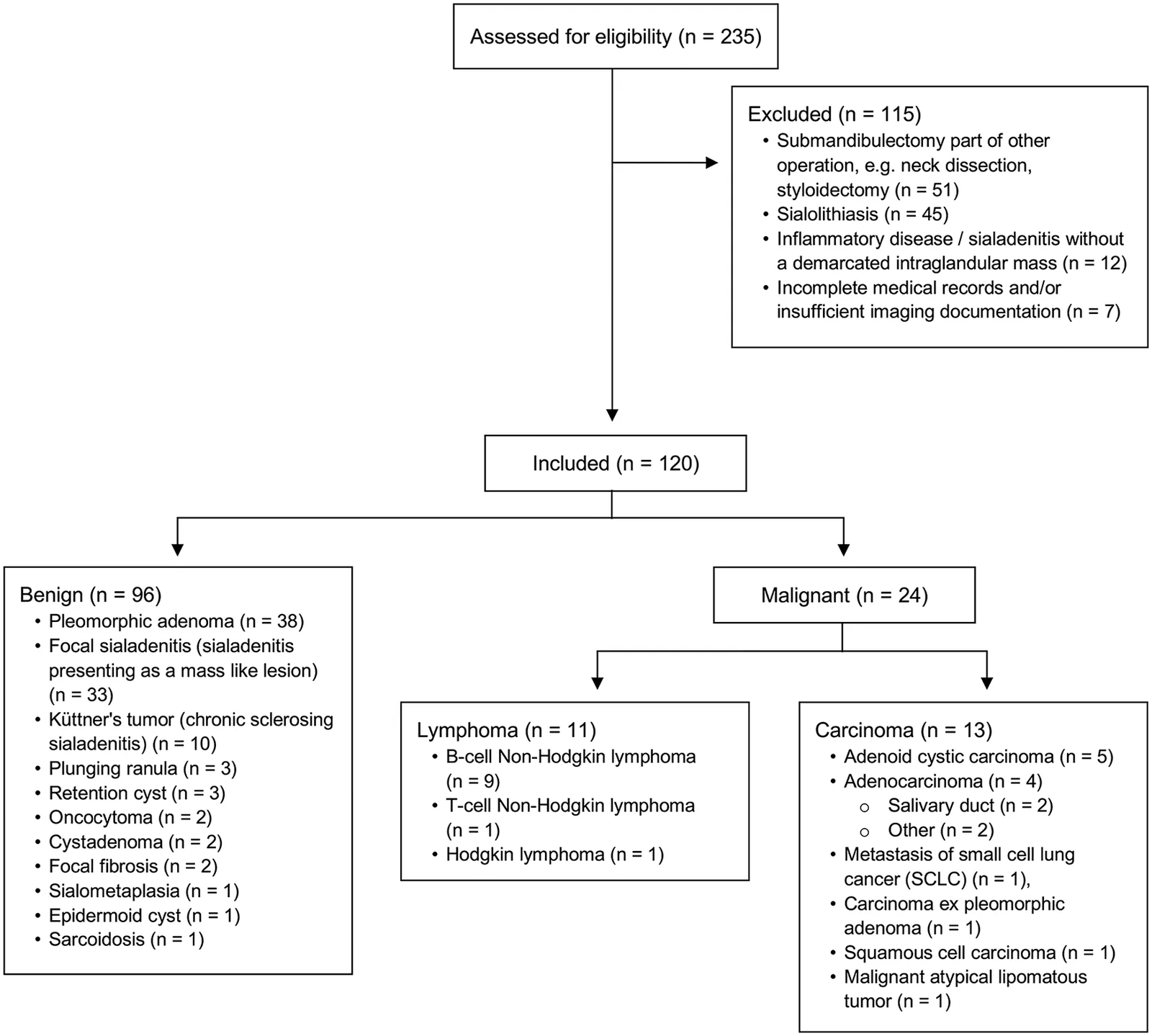
Patient selection flowchart and distribution of histologic subtypes. Of 235 patients assessed for eligibility, 120 patients were included in the study. Final histology revealed 96 benign lesions, 13 carcinomas, and 11 lymphomas.

We retrospectively reviewed all consecutive patients undergoing submandibulectomy for an intraglandular lesion at a high-volume tertiary university hospital between February 2019 and March 2025. Procedures performed for other indications, including sialolithiasis, inflammatory disease, or as part of a neck dissection for an extraglandular malignancy were excluded (Figure 1). All patients underwent a standardized preoperative ultrasound examination with a high-frequency linear transducer (PLT-1005BT, 10 MHz, Aplio i800 and Aplio a, Canon Medical Systems, Ōtawara, Japan) assessing the submandibular gland, the other major salivary glands, cervical lymph nodes, and the thyroid, with lesion documentation in two orthogonal planes. Color Doppler evaluation of vascularity was performed in most cases. Following resection, specimens were processed for histopathologic analysis, and demographic/clinical variables (age, sex, smoking status, and symptoms) were extracted from medical records.

All ultrasound images were re-evaluated by a single head and neck ultrasound examiner who was blinded to clinical and histopathologic outcomes. Tumor size was measured in three orthogonal dimensions, and the taller-than-wide (TTW) criterion was defined as a skin-perpendicular diameter exceeding the two remaining dimensions, with an additional TTW ratio calculated as the skin-perpendicular diameter divided by the largest diameter parallel to the skin, as previously described (29). Sonographic features including shape, margin characteristics, internal echotexture and echogenicity, posterior acoustic enhancement, calcifications, vascularity, multifocality, suspected skin invasion, and lymph node status were recorded. Salivary gland risk scores were calculated as previously published (28, 29).

Statistical analyses were performed in GraphPad Prism version 10.6.1 (GraphPad Software, Boston, MA, USA; RRID:SCR_002798). Normality was assessed using the Shapiro–Wilk test (*α* = 0.05). Descriptive statistics are reported as mean ± standard deviation (SD) for continuous variables and categorical variables are summarized as counts and percentages. The unpaired Student's t-test was used for parametric data, and nonparametric data were compared with the Mann–Whitney test. The Pearson's chi-square test was used for contingency analysis of categorical data. Subgroup analyses comparing three groups (benign, carcinoma, lymphoma) were performed using one-way analysis of variance (ANOVA). Significance was set at *p* < 0.05. Given the exploratory retrospective design, no *a priori* sample-size calculation was performed and no correction for multiple comparisons was performed, but individual *p* values are reported.

## 3 Results

### 3.1 Cohort characteristics

All 235 patients undergoing submandibulectomy between February 2019 and March 2025 were screened for eligibility. Those who had submandibulectomy as part of another operation, e.g., styloidectomy or neck dissection for a primary tumor not within the submandibular gland, patients with sialolithiasis and cases with incomplete medical records and/or insufficient imaging documentation were excluded. Patients with inflammatory changes of the entire submandibular gland without a demarcated lesion were excluded, as differential diagnosis of malignancy is rare in these cases, while those with a demarcated lesion were included.

Out of 120 cases in total, benign lesions were diagnosed in 96 (80.0%), including 38 (39.6%) patients with pleomorphic adenoma, 33 (34.4%) with focal sialadenitis (sialadenitis presenting as a mass-like lesion), 10 (10.4%) with Küttner tumor (chronic IgG4-positive sclerosing sialadenitis), three (3.1%) with retention cyst, two each (2.1%) with oncocytoma, cystadenoma, and focal fibrosis, and one case each (1.0%) of sialometaplasia, epidermoid cyst, and sarcoidosis. Three patients (3.1%), suspicious for intraglandular lesion of the submandibular gland after ultrasonographic assessment, were finally diagnosed with plunging ranula.

A carcinoma was diagnosed in 13 patients (10.8%), of which five (38.5%) had adenoid cystic carcinoma, four (30.7%) had adenocarcinoma, of which two (15.4%) were salivary duct carcinoma. One patient each (7.7%) had metastasis of small cell lung cancer (SCLC), carcinoma ex pleomorphic adenoma, squamous cell carcinoma, and malignant atypical lipomatous tumor. Lymphoma was diagnosed in 11 patients (9.2%). Mean age was 58.4 ± 17.4 years and there were 90 male patients (75.0%) and 30 female patients (25.0%).

### 3.2 Tumor morphology

**Figure 2:**
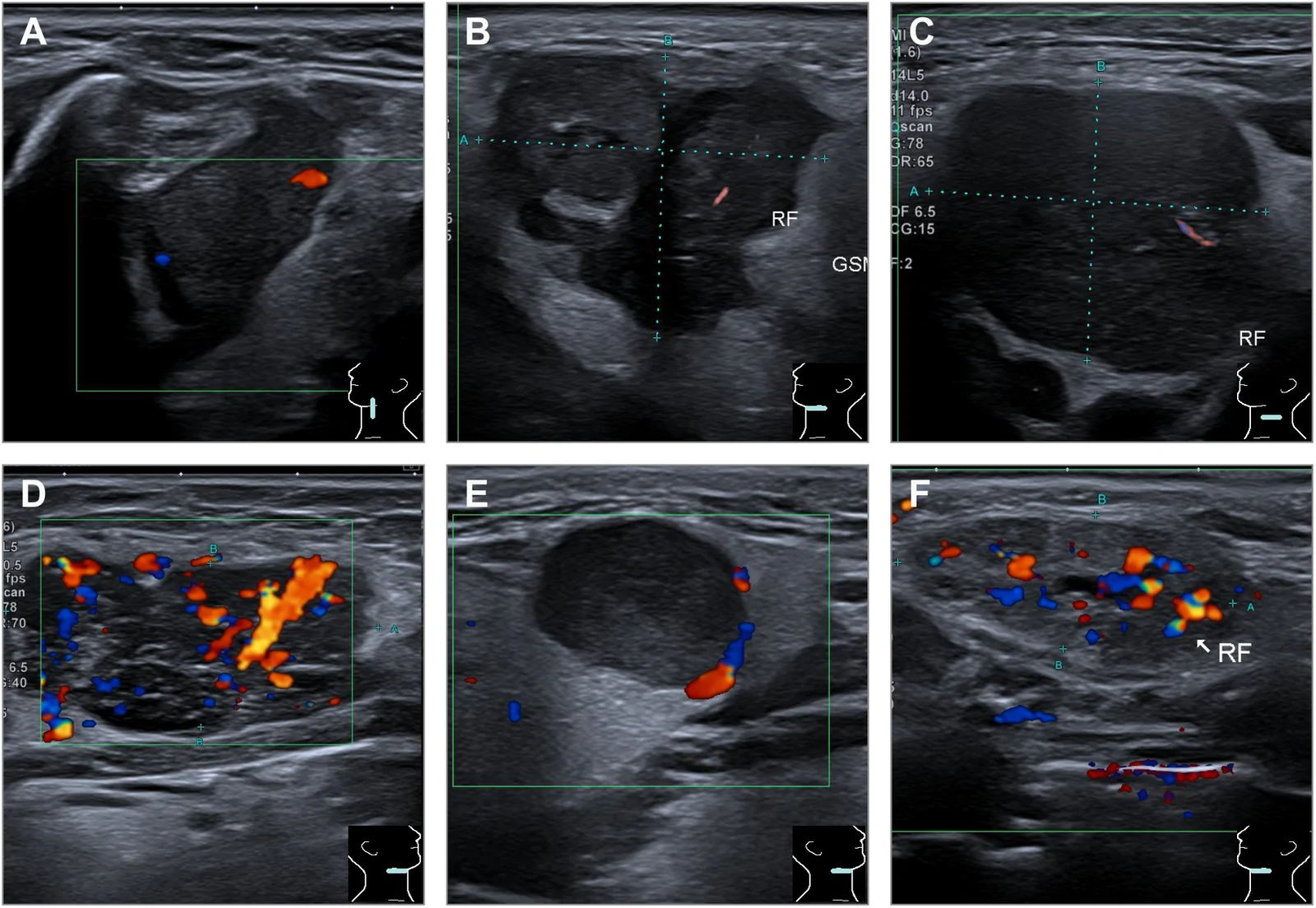
Color Doppler sonographic images (PLT-1005BT, 10 MHz, Aplio i800 and Aplio a, canon medical systems); added pictogram in the bottom right indicating scan plane and side. **(A)** Adenoid cystic carcinoma, **(B)** salivary duct carcinoma, **(C)** a highly suspicious cervical lymph node in level II of salivary duct carcinoma, **(D)** follicular intraglandular lymphoma (B-cell non-Hodgkin lymphoma), **(E)** pleomorphic adenoma, and **(F)** Küttner tumor (chronic IgG4-positive sclerosing sialadenitis).

In the qualitative assessment of morphology, most pleomorphic adenomas appeared as well-defined hypoechogenic tumors (Figure 2). Most had posterior acoustic enhancement and some were lobulated. Focal sialadenitis and Küttner tumor appeared as ill-defined areas within the gland, often with heterogeneous echotexture and increased vascularity. Adenoid cystic carcinomas were hypoechogenic, some homogeneous and some heterogeneous, with an irregular shape and some vascularity. The adenocarcinomas were either iso- or hypoechogenic, irregularly shaped with ill-defined margins.

### 3.3 Quantification of factors associated with malignancy

Out of 24 patients with a malignant tumor, one (4.2%) had an affected marginal mandibular branch of the facial nerve, compared to no affected nerves in the group with a benign lesion (*p* = 0.04). The other clinical features – including age, sex, active smoking, pain, and B symptoms – showed no significant difference between the groups (all *p* > 0.05, Table 1). Malignant tumors more frequently had irregular margins (9 (37.5%) vs. 17 (17.7%), OR 2.78, 95%-CI 1.09–7.41, *p* = 0.04) and sonographically suspicious cervical lymph nodes (5 (20.8%) vs. 1 (1.0%), OR 25.00, 95%-CI 2.96–296.4, *p* = 0.001). There were no significant differences in the other ultrasound features, including tumor size, long-to-short axis ratio, multifocality, non-homogeneous appearance, avascularity, hypoechogenic appearance, posterior acoustic enhancement, and calcification (all *p* > 0.05, Table 2).

**Table 1:** Clinical features of patients presenting with a benign or malignant lesion of the submandibular gland.

Clinical feature	Benign (*n* = 96)	Malignant (*n* = 24)	*p* value
Age (mean ± SD, years)	57.4 ± 18.3	63.5 ± 13.1	0.15
Female sex (No., %)	23 (24.0%)	7 (29.2%)	0.59
Active smoking (No., %)	10 (10.4%)	2 (8.3%)	0.76
Pain (No., %)	17 (17.7%)	3 (12.5%)	0.54
B symptoms (No., %)	2 (2.1%)	2 (8.3%)	0.13
Facial nerve palsy (No., %)	0 (0%)	1 (4.2%)	**0** **.** **04**

Bold values indicate statistical significance (*p* < 0.05).

**Table 2:** Ultrasound criteria for tumors defined as benign or malignant in final histology.

Ultrasound feature	Benign (*n* = 96)	Malignant (*n* = 24)	*p* value
Tumor size (mean ± SD, mm)	21.5 ± 8.4	24.7 ± 8.1	0.05
Long-to-short axis ratio (mean ± SD)	1.69 ± 0.55	1.65 ± 0.54	0.54
Taller-than-wide ratio (mean ± SD)	0.65 ± 0.19	0.70 ± 0.26	0.45
Multifocality ipsi- or contralateral (No., %)	6 (6.3%)	1 (4.2%)	0.70
Contralateral tumor (No., %)	1 (1.0%)	0 (0%)	0.62
Irregular margins (No., %)	17 (17.7%)	9 (37.5%)	**0** **.** **04**
Infiltration of skin (No., %)	0 (0%)	0 (0%)	–
Non-homogenous appearance (No., %)	52 (54.2%)	14 (58.3%)	0.71
Avascular in color Doppler sonography^a^ (No., %)	16 (23.5%)	3 (17.6%)	0.60
Hypoechogenic (No., %)	59 (61.5%)	17 (70.8%)	0.39
Posterior acoustic enhancement (No., %)	42 (43.8%)	13 (54.2%)	0.36
Calcification (No., %)	4 (4.2%)	2 (8.3%)	0.40
Suspicious cervical lymph nodes (No., %)	1 (1.0%)	5 (20.8%)	**0**.**001**

a Color Doppler sonography was done in 85 patients, 68 with benign and 17 with malignant lesions.

b Bold values indicate statistical significance (*p* < 0.05).

### 3.4 Applicability of salivary gland risk scores to the submandibular gland

Multiple risk factors and scores for salivary gland lesions were assessed. There were no significant differences concerning the taller-than-wide ratio between malignant and benign lesions (0.70 ± 0.26 vs. 0.65 ± 0.19, median difference 0.04, 95%-CI −0.06–0.13, *p* = 0.45). Subgroup analysis using one-way ANOVA showed a difference (*p* = 0.02) between the groups of carcinomas (0.80 ± 0.28), lymphomas (0.59 ± 0.17), and benign lesions (0.65 ± 0.19). *post hoc* analysis showed significant differences between carcinoma and benign tumors and between carcinoma and lymphoma.

The score proposed by Lo et al. (28), which is calculated as follows: 2.08 × (boundary) + 1.75 × (regional lymphadenopathy) + 1.18 × (shape) + 1.45 × (posterior acoustic enhancement) + 2.4 × (calcification), showed no significant differences between malignant (2.30 ± 2.16) and benign lesions (1.46 ± 1.58, *p* = 0.054). ROC analysis revealed an area under the ROC curve (AUC) of 0.62 (95%-CI 0.48–0.76), indicating a poor discriminatory ability of the score. The modification of this score for parotid gland tumors proposed by our research group (29) included an additional dichotomized TTW ratio, defined as positive when the TTW ratio was ≥ 0.82 and weighted 1.5. This threshold of 0.82 corresponds to the cut-off that maximized the Youden index in the initial parotid gland cohort, where it yielded a sensitivity of 50.0% and a specificity of 80.5% for malignancy (AUC of the TTW ratio alone, 0.66) (29). This score also showed no significant differences between malignant (2.62 ± 2.46) and benign lesions (1.70 ± 1.63, *p* = 0.10) and an AUC of 0.61 (95%-CI 0.47–0.74) indicated poor discriminatory ability.

## 4 Discussion

In this retrospective surgical cohort of submandibular gland mass lesions, preoperative ultrasound showed limited ability to distinguish benign from malignant disease based on morphology alone. Among the assessed parameters, only irregular margins and sonographically suspicious cervical lymph nodes were significantly associated with malignancy, aligning with the existing literature for salivary gland in general (30, 31) and submandibular glands (32), while most other clinical and sonographic features did not differ meaningfully between groups.

These results contrast with prior work in major salivary gland cohorts and especially in the parotid gland, where ultrasound-based malignancy prediction has shown more promising performance. Notably, the score proposed by Lo et al. was derived predominantly from parotid tumors, with only a minority of submandibular lesions included, and was internally validated in a similarly mixed cohort (28, 33). We previously found that this model performed well in a parotid-only cohort and that adding a dichotomized TTW criterion further improved diagnostic accuracy (29). In the present study, however, neither the original nor the modified score achieved clinically useful discrimination. This suggests that risk models based on pooled major salivary gland populations should not be assumed to generalize across glands without dedicated external validation.

Several factors may explain the limited transferability of these models to the submandibular gland. First, local anatomy differs substantially between the parotid and submandibular spaces. The submandibular gland has a more compact, rounded configuration and is bordered by the mandible, mylohyoid muscle, digastric muscle, and floor of mouth, which likely constrains tumor expansion differently from the parotid region. As a consequence, a skin-perpendicular growth pattern may be less reflective of infiltrative biology in the submandibular gland than in the parotid gland. Second, the case mix differs. Unlike *post hoc* analyses limited to resected submandibular tumors, our lesion-centered approach was designed to reflect the real preoperative ultrasound setting, in which inflammatory disease may also present as a demarcated intraglandular lesion. Accordingly, a substantial proportion of surgically treated benign lesions in our cohort were inflammatory tumor-mimicking processes, including focal sialadenitis and Küttner tumor, as these are more common and more frequently treated surgically than in the parotid gland (34–36). Such lesions may share ill-defined margins, heterogeneous echotexture, and altered vascularity with malignant tumors, thereby reducing the specificity of morphology-based scores. Third, the malignant group itself was heterogeneous and included both carcinomas and lymphomas, which are biologically distinct entities with different expected sonographic appearances and management pathways.

The taller-than-wide (TTW) criterion deserves particular comment. In thyroid (37, 38), breast (39), and more recently parotid ultrasound (29), TTW orientation has been linked to malignant growth across normal tissue planes. In the present submandibular cohort, however, the continuous TTW ratio was not significantly different between benign and malignant lesions overall, although an exploratory subgroup analysis suggested that carcinomas may differ from lymphomas and benign lesions. This finding should be interpreted cautiously given the small subgroup sizes, but it raises the possibility that TTW may capture only selected malignant phenotypes rather than malignancy *per se* in the submandibular gland. Methodological issues may also contribute: compared with the parotid region, the submandibular gland is more obliquely positioned, lesion depth is more variable, and probe angulation or transducer compression may affect measurement of the perpendicular axis, potentially reducing reproducibility of TTW-based metrics.

Several limitations must be considered when interpreting these findings. The retrospective single-center design of a surgical cohort introduces the possibility of selection and information bias. As submandibular gland neoplasms are rare, the number of malignant lesions was small in our cohort, reducing statistical power, increasing the risk of type II error and making subgroup analyses challenging. In particular, tumor size (*p* = 0.05) and the Lo et al. score (*p* = 0.054) approached but did not reach statistical significance, and for these parameters a true association that the study was underpowered to detect cannot be excluded. Only surgical cases were included in our cohort, as final histology was used to assess the ultrasound images. Consequently, the study population may not represent the full spectrum of submandibular gland lesions encountered in clinical practice, limiting generalizability to non-surgical cohorts. Distinguishing a genuinely intraglandular pathology from peri-glandular nodal or extraglandular pathology can be challenging. The study cohort was defined by lesions presenting as demarcated intraglandular masses, which does not encompass all pathologies encountered in the submandibular region.

The malignant and benign groups were histologically heterogeneous, which may obscure associations that are present only within specific entities. This heterogeneity is likely to have attenuated the overall benign-versus-malignant comparison. The taller-than-wide ratio was highest in carcinomas (0.80 ± 0.28) and lowest in lymphomas (0.59 ± 0.17), with benign lesions intermediate (0.65 ± 0.19), and the carcinoma subgroup differed significantly from both benign lesions and lymphomas. Pooling these two divergent phenotypes into a single malignant category therefore averages opposing signals and dilutes the apparent association between malignancy and individual sonographic features. Because of the exploratory nature of the study, no correction for multiple testing was applied and *p* values should be interpreted accordingly.

From a clinical perspective, our findings reinforce a pragmatic role for ultrasound in the work-up of submandibular gland lesions. Ultrasound is generally well suited for identifying the intraglandular origin of a lesion (21), assessing cervical lymph nodes (40–42), and identifying overtly suspicious features such as irregular margins or nodal disease. However, the substantial overlap in sonographic appearance between benign neoplasms, inflammatory mass lesions, carcinoma, and lymphoma indicates that ultrasound morphology alone should not be used as a stand-alone rule-in or rule-out test for malignancy. This highlights the necessity for tissue diagnosis, e.g., by fine-needle aspiration cytology (23, 24), core needle biopsy (25), intraoperative frozen-section pathology (27, 43), or postoperative histopathologic evaluation (44, 45).

An MRI should be performed in cases where malignancy is suspected, because it provides objective cross-sectional documentation prior to treatment planning and may detect malignant features not identifiable by other methods, particularly perineural invasion (46–48). For distinguishing between benign and malignant salivary gland tumors with MRI, pooled sensitivity is 80% and specificity is 90%, while specific data for the submandibular gland are scarce (46). Advanced MRI techniques including diffusion-weighted imaging (DWI) with ADC mapping and dynamic contrast-enhanced (DCE) MRI with time-intensity curve analysis should be considered (47, 49, 50). CT with contrast should be added if osseous invasion is suspected (15, 47, 51).

Improvements in sonography techniques might enhance the diagnostic accuracy of preoperative ultrasound in submandibular gland tumors. Elastography has emerged as a valuable adjunctive imaging modality (52, 53), demonstrating superior performance compared to qualitative methods and achieving pooled sensitivity of 73% and specificity of 64% for differentiating benign from malignant tumors (54), despite substantial overlap in stiffness values between benign and malignant lesions (55, 56). Contrast-enhanced ultrasound might also improve accuracy and blurred enhancement margins and increased lesion size after enhancement in particular are associated with malignancy (57, 58).

In conclusion, preoperative ultrasound remains an essential component of the diagnostic pathway for submandibular gland mass lesions, but its capacity for morphology-based discrimination between benign and malignant disease appears limited in a real-world surgical cohort and therapeutic decisions must be based on some form of tissue diagnosis – frozen-section pathology, fine-needle aspiration cytology or core needle biopsy depending on local preconditions. Irregular margins and suspicious cervical lymph nodes were the most informative sonographic findings, whereas parotid-derived sonographic risk scores, including TTW-based modification, showed poor transferability to the submandibular gland. Future multicenter studies should aim to develop submandibular-specific prediction models that integrate ultrasound features with cytology, cross-sectional imaging, and clinical variables.

## Data Availability

The datasets presented in this article are not readily available because of patient privacy and confidentiality restrictions. De-identified data supporting the findings of this study are available from the corresponding author upon reasonable request and subject to approval by the local ethics committee. Requests to access the datasets should be directed to AS, andreas.spoerlein@uniklinik-freiburg.de.
